# Consistency, comprehensiveness, and compatibility of pathway databases

**DOI:** 10.1186/1471-2105-11-449

**Published:** 2010-09-07

**Authors:** Donny Soh, Difeng Dong, Yike Guo, Limsoon Wong

**Affiliations:** 1 School of Computing, National University of Singapore, Building COM1, 117417 Singapore; 2 Department of Computing. Imperial College London. 180 Queen's Gate. London SW7 2BZ, UK; 3 Institute for Infocomm Research, 1 Fusionopolis Way, 138632 Singapore

## Abstract

**Background:**

It is necessary to analyze microarray experiments together with biological information to make better biological inferences. We investigate the adequacy of current biological databases to address this need.

**Description:**

Our results show a low level of consistency, comprehensiveness and compatibility among three popular pathway databases (KEGG, Ingenuity and Wikipathways). The level of consistency for genes in similar pathways across databases ranges from 0% to 88%. The corresponding level of consistency for interacting genes pairs is 0%-61%. These three original sources can be assumed to be reliable in the sense that the interacting gene pairs reported in them are correct because they are curated. However, the lack of concordance between these databases suggests each source has missed out many genes and interacting gene pairs.

**Conclusions:**

Researchers will hence find it challenging to obtain consistent pathway information out of these diverse data sources. It is therefore critical to enable them to access these sources via a consistent, comprehensive and unified pathway API. We accumulated sufficient data to create such an aggregated resource with the convenience of an API to access its information. This unified resource can be accessed at http://www.pathwayapi.com.

## Background

It is challenging to draw biological conclusions from today's microarray experiments. The main source of the difficulty is that the number of samples available for analysis is usually very small relative to the number of genes to be considered. It is often the case that many genes are statistically significant according to the wide variety of computational and statistical analysis algorithms. Yet there is little concurrence between the genes selected by different algorithms. Furthermore, the genes selected by these algorithms do not always provide an insight that is biologically consistent or biologically interpretable.

In order to obtain results that are more biologically meaningful, it is important to incorporate information from biological repositories into the analysis of microarray data [1]. Indeed, most of the new generation of algorithms incorporate information from biological pathways into microarray data analysis [2-4].

Examples of the new generation of microarray data analysis algorithms that incorporate biological pathway information into the analysis process include ORA (Over Representation Analysis) [4,5], FCS (Functional Class Scoring) [4,5], GSEA (Gene Set Enrichment Analysis) [3], ErmineJ [6] and Pathway Express [7].

Examples of databases which these algorithms reference are: NCBI [8], KEGG [9-11], Ingenuity [12], GO (Gene Ontology) [13] and Wikipathway [14]. In terms of source authority, both KEGG and Ingenuity derive their data from published work while Wikipathways first derive their's from several established databases (eg KEGG, Netpath) and are subsequently curated by the research community.

However these biological databases are very diverse, making it extremely laborious to carry out even simple queries across databases. To make matters worse, inconsistencies and incompatibilities between different repositories render the individual databases less effective for collaborative purposes.

This inconsistency is worsened because the boundaries of signaling pathways are not that clearly defined scientifically. For example, the pathway "MAPK Cascade" probably has no clear consistent definitions in the literature hence making the question of exactly which genes to include quite subjective [15].

ORA, FCS and GSEA are all examples of algorithms that incorporate information from biological databases. Both ORA and FCS use the GO database to select relevant genes according to their GO classes. GSEA uses their proprietary database (curated from various sources) for gene selection.

The importance of the accuracy and comprehensiveness of the biological pathway information used should be clear from the short review above of modern microarray data analysis algorithms. For instance, clinicians may potentially end up with different results and conclusions depending on the database they group their genes by!

Therefore we study the following issues in this paper:

+ Are various selected biological pathway data sources consistent with each other?

+ Are they sufficiently comprehensive individually?

+ Are the databases easily accessible to researchers who wish to use their data for their analysis?

Although there are many commonly used pathway databases (eg NCBI, GO, Reactome, HumanCyc, BIGG, Panther Pathways, Science STK, etc) we have selected three data sources (KEGG, Ingenuity and Wikipathways) for our analysis. These sources are chosen because they are representative of three very different kinds of curation effort. For instance, Wikipathways is maintained by a community of professional users via the free and open wiki platform. KEGG database is curated independently by a single lab from published literature. Ingenuity is a commercial product.

Our results show a low level of consistency, comprehensiveness and compatability among these three selected pathway databases. We addressed these issues with a unified easy-to-use API which allows access to biological pathway information from KEGG, Ingenuity, and Wikipathways. This common API allows researchers to gain instant and updated access to data from the different repositories.

## Results

### Database Consistency

Pathway databases (eg KEGG, Ingenuity, Wikipathways) have always been assumed to be consistent because they share a common data source: published literature (Wikipathways is based on established databases like KEGG or Netpath, hence sharing the same roots of published literature). We show here that this assumption is not true.

We define the following metrics to illustrate the diversity across databases. The first metric, the "Gene Agreement Count" of a pathway, counts the number of genes that are common to that pathway in all the databases. The second metric, the "Gene Pair Agreement Count" of a pathway counts the number of "interacting gene pairs" that are common to that pathway in all the databases. An interacting gene pair is a pair of genes (or their products) that are directly interacting in a pathway. In the case of metabolic pathways, however, we define an interacting gene pair as proteins that catalyze adjacent steps in the pathway.

When calculating the "Gene Agreement Percentage" of a pathway, we first find the total number of genes within that pathway for each individual database. We next select the gene count from the database that has the least number of genes for that pathway. Finally we divide the Gene Agreement Count by this mininum gene count to obtain the Gene Agreement Percentage. The same technique is employed to calculate the Gene Pair Agreement Percentage.

The three databases represent some of their pathway entries not as genes but as proteins or symbols depicting protein families or classes. In such instances we replace all such proteins and symbols with the genes they represent. For example, suppose that A activates B within a pathway, where A and B are symbols representing protein classes that are products of 3 genes and 2 genes respectively. We replace A activates B by 6 new activating relationships. We claim the validity of this replacement method because it exactly captures all the genes and relationships the original curator had intended. All statistics calculated here are based on the expanded relationships.

Our investigation into database consistency began with a manual comparison on the agreement of the apoptosis pathway across databases. Results indicate a range of 11%-16% (Gene Pair Agreement Percentage) and 32%-46% (Gene Agreement Percentage). This is an extremely low level of agreement given a pathway as pervasive as the apoptosis pathway. Full results are seen in Table 1.

**Table 1:** Table showing data overlap for Apoptosis Pathway

Apoptosis Pathway			
	**KEGG × Ingenuity**	**KEGG × Wiki**	**Ingenuity × Wiki**

Gene Pair Count:	151 vs 3374	151 vs 133	3374 vs 133

Gene Count:	89 vs 169	89 vs 82	169 vs 82

Gene Overlap:	33	38	26

Gene % Overlap:	37%	46%	32%

Gene Pair Overlap:	21	21	15

Gene Pair % Overlap:	14%	16%	11%

This table shows the manual calculation of the gene/gene pair differences between the different repositories for the apoptosis pathway.

The next step involved an automated extraction for the apoptosis pathway between the databases. The results are shown in Table 2. The results indicate a range of 12%-14% (Gene Pair Agreement Percentage) and 30%-46% (Gene Agreement Percentage). This is indicative that the above-mentioned automatic extraction and gene matching procedure is reliable and not missing significant numbers of equivalent genes. We subsequently followed up with an automated extraction and comparison between the databases. The ranges are 0%-88% (Gene Agreement Percentage) and 0%-61% (Gene Pair Agreement Percentage). These numbers comfirm our earlier suspicion that there is an extremely low level of consistency between the databases. For results depicting the level of overlap for the other pathways refer to Tables 3, 4 and 5.

**Table 2:** Table showing data overlap for Apoptosis Pathway

Apoptosis Pathway			
	**KEGG × Ingenuity**	**KEGG × Wiki**	**Ingenuity × Wiki**

Gene Pair Count:	182 vs 3486	182 vs 155	3486 vs 155

Gene Count:	84 vs 185	84 vs 79	185 vs 79

Gene Overlap:	28	36	24

Gene % Overlap:	33%	46%	30%

Gene Pair Overlap:	22	22	18

Gene Pair % Overlap:	12%	14%	12%

This table shows the calculation of the gene/gene pair differences between the different repositories for the apoptosis pathway based on the automated processing described in this paper.

**Table 3:** Table showing data overlap between KEGG × Ingenuity

KEGG × Ingenuity				
**Pathway Name**	**Gene Count**	**Pair Count**	**Gene % Overlap**	**Pair % Overlap**

Apoptosis Signaling	89 vs 169	151 vs 3374	33(37%)	21(14%)

Axonal Guidance	129 vs 213	308 vs 1843	85(66%)	159(52%)

Calcium Signaling	179 vs 51	582 vs 202	18(35%)	0(0%)

Cell Cycle-G2M	119 vs 13	78 vs 18	11(85%)	11(61%)

Cell cycle	119 vs 31	78 vs 59	26(84%)	6(10%)

Fc epsilon RI signaling	78 vs 75	184 vs 225	61(81%)	108(59%)

JAK/Stat Signaling	155 vs 144	868 vs 3192	42(29%)	88(10%)

Actin Cytoskeleton Signaling	217 vs 213	672 vs 2297	137(64%)	230(34%)

T cell receptor Signaling	94 vs 63	175 vs 133	41(65%)	39(29%)

TGF-Beta Signaling	87 vs 84	155 vs 113	12(14%)	5(4%)

VEGF Signaling	74 vs 69	240 vs 167	29(42%)	27(16%)

Wnt Signaling	152 vs 76	778 vs 134	33(43%)	11(8%)

**Table 4:** Table showing data overlap between KEGG × Wiki

KEGG × Wiki				
**Pathway Name**	**Gene Count**	**Pair Count**	**Gene % Overlap**	**Pair % Overlap**

Apoptosis	89 vs 82	151 vs 133	38(46%)	21(16%)

Apoptosis Modulation by HSP70	89 vs 18	151 vs 33	14(78%)	5(15%)

Cell cycle	119 vs 91	78 vs 147	76(84%)	35(45%)

G1 to S cell cycle control	119 vs 67	78 vs 25	45(67%)	1(4%)

Complement and coagulation cascades	69 vs 31	69 vs 107	52(80%)	24(35%)

Focal Adhesion	203 vs 188	706 vs 288	154(82%)	110(38%)

Insulin Signaling	138 vs 159	412 vs 255	66(48%)	13(5%)

MAPK Cascade	269 vs 31	819 vs 55	23(74%)	24(44%)

Notch Signaling	46 vs 46	90 vs 98	39(85%)	32(36%)

Regulation of actin cytoskeleton	217 vs 151	672 vs 244	133(88%)	113(46%)

T Cell Receptor Signaling	94 vs 135	175 vs 261	37(39%)	6(3%)

TGF Beta Signaling	87 vs 52	155 vs 80	23(44%)	6(8%)

Tryptophan metabolism	51 vs 94	233 vs 33	29(57%)	2(6%)

Urea cycle	28 vs 66	69 vs 14	13(46%)	1(7%)

Wnt signaling	152 vs 61	778 vs 184	49(80%)	34(18%)

**Table 5:** Table showing data overlap between Ingenuity × Wiki

Ingenuity × Wiki				
**Pathway Name**	**Gene Count**	**Pair Count**	**Gene % Overlap**	**Pair % Overlap**

Apoptosis	169 vs 82	3374 vs 133	26(32%)	15(11%)

Calcium Signaling	51 vs 152	202 vs 111	14(27%)	0(0%)

Cell Cycle	13 vs 91	18 vs 147	7(54%)	5(28%)

G1/S Check point Regulation	31 vs 91	59 vs 147	24(77%)	10(17%)

IL-4 Signaling	21 vs 62	21 vs 47	8(38%)	1(5%)

IL6 Signaling	67 vs 100	148 vs 121	21(31%)	4(3%)

Insulin Recpetor Signaling	66 vs 159	148 vs 255	40(61%)	12(8%)

TGF-Beta Signaling	84 vs 52	113 vs 80	13(25%)	0(0%)

p38 MAPK Signaling	53 vs 34	88 vs 35	13(38%)	4(11%)

T cell receptor Signaling	63 vs 135	133 vs 261	25(40%)	3(2%)

Wnt Signaling	76 vs 61	134 vs 184	17(28%)	0(0%)

### Pathway Consistency

The pathways across separate databases were matched via the following longest-common-substring-based technique (LCS): Given a pathway × in database 1, we generate a list of pathways Y in database 2. This list Y is ranked according to the length of the longest common substrings with pathway X. This list is next manually scanned to obtain the pathway which has the closest nomenclatural match to pathway X.

We compare three possible algorithms which could have been used to find pathway matches and they are

1. LCS-based algorithm: pathways are matched by matching the names of the pathways to the pathway with the closest name followed by a manual verification.

2. Gene pair overlap: pathways are matched by finding pairs of pathways with the maximum number of matched interacting gene pairs.

3. Gene overlap: pathways are matched by finding pairs of pathways with the maximum number of matched genes.

We carry out experiments to determine which of the three approaches above is most suitable for finding the best pathway matches. The quality of these algorithms can be judged according to two different aspects:

1. The percentage accuracy of matching pathways found: how accurate are the matching pathways found by each individual algorithm (finding the maximum number of true positives).

2. The completeness of the algorithm in finding all matching pathways: is the algorithm able to match up all pathways that should be matched and not pair up pathways that should not be matched (finding the minimum number of false negatives).

Intuitively, the pathway pairs based on gene overlap would have a lower accuracy than that of the gene pair based overlap. The reason being it is much easier to have spurious overlaps in genes as compared to gene pairs. Therefore there will be a greater number of false positives using the gene overlap algorithm. Hence we perform quantitative analysis mainly on the LCS and gene pair algorithms.

**Figure 1:**
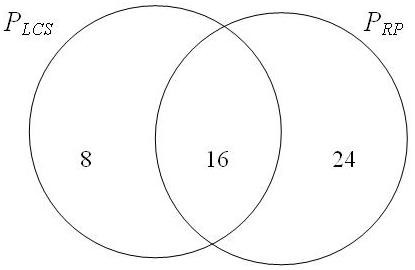
**Comparison of pathway pair overlaps between the LCS and gene pair overlap algorithm**. Venn diagram showing the pathway matches which are similar and between the LCS and gene pair overlap algorithm.

Let the matched pathway pairs found by the LCS algorithm be defined as *P_LCS _*and the matched pathway pairs found by the gene pair algorithm be defined as *P_RP _*. For the sake of comparison (Figure 1), in both cases, we include only those pathway pairs that have an overlap of at least 20 interacting gene pairs. (If a lower threshold was used, *P_RP _*would have an alarming increase in false positives).

In total, LCS produces 24 pathway pairs while the gene overlap algorithm produces 40 pathway pairs. 16 pathway pairs are common among these two algorithms so they naturally do not affect any comparative studies between the two algorithms. We thus ignore them in this analysis. The 8 pathway pairs exclusively singled out by our LCS technique are seen in Table 6 and the 14 pathway pairs exclusively singled out by the gene pair technique are seen in Table 7.

**Table 6:** Table comparing pathway pairs obtained from the LCS algorithm.

Pathway 1	Pathway 2
Regulation of actin cytoskeleton	Regulation of Actin Cytoskeleton

Wnt signaling pathway	Wnt Signaling Pathway

T cell receptor signaling	T cell receptor Signaling

VEGF signaling	VEGF Signaling

MAPK signaling	MAPK Cascade

Apoptosis	Apoptosis

Apoptosis	Apoptosis Signaling

Toll-like receptor	Toll-like receptor signaling pathway

**Table 7:** Table comparing pathway pairs obtained from the gene pair overlap algorithm.

Pathway 1	Pathway 2
ErbB signaling pathway	JAK/Stat Signaling

Calcium signaling pathway	Synaptic Long Term Potentiation

Apoptosis	Toll-like receptor signaling pathway

VEGF signaling pathway	Axonal Guidance Signaling

Gap junction	PPAR-alpha/RXR-alpha Signaling

Natural killer cell mediated cytotoxicity	Fc Epsilon RI Signaling

T cell receptor signaling pathway	Axonal Guidance Signaling

B cell receptor signaling pathway	Axonal Guidance Signaling

Olfactory transduction	cAMP-mediated Signaling

GnRH signaling pathway	B Cell Receptor Signaling

Melanogenesis	Wnt Signaling Pathway and Pluripotency

Type II diabetes mellitus	Insulin Recpetor Signaling

Colorectal cancer	Toll-like receptor signaling pathway

Renal cell carcinoma	Axonal Guidance Signaling

Pancreatic cancer	PTEN Signaling

Endometrial cancer	PTEN Signaling

Glioma	ERK/MAPK Signaling

Prostate cancer	JAK/Stat Signaling

Basal cell carcinoma	Wnt Signaling Pathway and Pluripotency

Melanoma	FGF Signaling

Chronic myeloid leukemia	GM-CSF Signaling

Acute myeloid leukemia	PTEN Signaling

Small cell lung cancer	Toll-like receptor signaling pathway

Non-small cell lung cancer	GM-CSF Signaling

Comparing Table 6 and Table 7, we see that the pathway pairs in Table 6 are much more reasonable than that of the pathway pairs in Table 7. This shows that the LCS technique has a higher sensitivity in detecting true positives. In addition, the rate of false negatives is also lower because, even at a cursory look, none of the pairs found by the gene-pair-based overlap matching technique can be true matches in pathway pairs.

An example of a false positive found by comparing gene pairs is the pathway pair "Long-term potentiation (LTP)" and "Calcium signaling pathway" (Second line in Table 7). Long-term potentiation (LTP) is the increase of synaptic strength between two neurons following high frequency stimulation to the synapse. It occurs when the concentration of calcium inside the postsynaptic cell exceeds a critical threshold. A majority of synapses that experience LTP involve an increase in calcium which is mediated through activation of the NMDA receptor. The difference is that the Calcium signaling pathway in KEGG describes the general mechanism of external calcium signal transduction into cells. This process may take place via multiple pathways, and the NMDA receptor is only one of them. Nevertheless, the calcium signal transduction can actually activate multiple downstream pathways, and LTP is one of them. Thus, the LTP in Ingenuity can be considered as only a downstream event of the calcium pathway in KEGG.

Another example is that of the pathway pair between PPAR-alpha and TGF-beta (not in top list). PPAR-alpha is a ligand activated transcription factor that belongs to the family of nuclear receptors. After binding with its partner RXR-alpha, PPAR-alpha plays essential roles in the regulation of cellular differentiation, development, metabolism, and tumorigenesis of higher organisms. On the other hand, TGF-beta acts as antiproliferative factor in normal cells at early stages of oncogenesis. It phosphorylates smad2/3, which consequently binds with smad4 to form an antitumorigenesis transcription factor. The formed smad2/3-smad4 factor is mutually inhibited with PPAR-alpha-RXR-alpha complex. Thus the two pathways are independent since both factors are not involved in the key processes of the other pathway. The reason they are paired is that they may have a mutual inhibition.

Furthermore, we realize that because some pathway pairs that should be matched have low levels of gene pair overlap. Consequently the gene-pair-based matching technique is unable to match them (without simultaneously introducing a high level of false positive matches). An example of such pathway pairs is the TGF-Beta Signaling pathway between KEGG and Ingenuity (gene pair overlap ≤ 4%), Wnt Signaling (gene pair overlap ≤ 8%) and Cell cycle (gene pair overlap ≤ 10%). These pathway pairs however are successfully matched by our LCS technique thus providing us with more opportunities to merge and reconcile data resulting in pathways that are more complete.

For the sake of completeness of analysis, we perform three further checks that compare the LCS, gene-overlap and gene-pair-overlap methods.

#### Comparison between LCS and gene overlap algorithms

We carry out a comparison between the LCS algorithm and the gene overlap algorithm via the following technique.

1. For each pathway *P*_1_, find a ranked list of pathways PL such that each pathway *PL_i _*within PL is ranked according to the gene overlap between *P*_1 _and *PL_i_*.

2. Execute the LCS algorithm on pathway *P*_1 _and obtain its matching pathway, *P*_*LCS*1_.

3. Compare the ranked list PL with pathway *P*_*LCS*1_.

4. 94% of the time, the pathway *P*_*LCS*1 _obtained using the LCS algorithm is found in the top three pathways within *PL*. The LCS algorithm disagrees with the gene-overlap algorithm on the remaining 6% of pathways. These 6% of pathways have a large number of genes. Leading to a higher probability of spurious overlapping of genes.

5. To confirm, for these remaining 6% of pathways, we calculate the percentage gene pair overlap between *P*_1 _and *P_LCS1 _*and that of *P*_1 _and the top pathways in *PL*. It turns out that the former is always higher than that of the top three pathways in *PL*. This shows that the accuracy of the LCS algorithm is sufficiently good.

#### Comparison between LCS and gene pair overlap algorithm

We carry out a second comparison between the LCS algorithm and the gene pair overlap algorithm via the following technique.

1. Compile a list of pathways *PU *that the LCS algorithm could not find a match.

2. For each of the pathways *PU_i _*on this list, find a corresponding matching pathway *PUM_i _*by using the pathway with the highest gene overlap percentage.

3. We calculate the gene pairs percentage overlap between each pathway pair *PU_i _*and *PUM_i_*.

4. Similarly, for each pathway *P*_1 _that the LCS algorithm could find a matching pathway *P*_*LCS*1_, we calculate the gene pairs percentage overlap between pathway *P*_1 _and its matching pathway *P*_*LCS*1_.

5. The percentage overlap found in Step (3) is generally significantly lower than that in Step (4). Evidently, our LCS technique manages to match up pathways that should be matched and does not pair up those that should not be matched.

**Figure 2:**
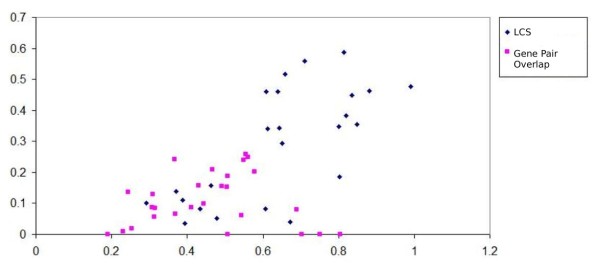
**Comparison of gene/gene pair overlap on matching pathways**. Image comparing the percentage of gene and gene pair overlap between matching pathways obtained from the LCS algorithm (in blue) and that of the gene overlap algorithm (in pink). The pink marks refer to pathways which are unable to be paired by the LCS algorithm. The x-axis depicts the gene overlap percentage and the y-axis the gene pair overlap percentage. The image shows that pathways matched by the LCS algorithm consistently have a higher gene pair overlap percentage.

This is illustrated in Figure 2. The blue spots indicate the pathway pairs found by the LCS algorithm. The purple spots indicate the pairs found by the gene pair overlap algorithm (that the LCS algorithm is unable to find a match for). The x-axis refers to the percentage of gene overlap while the y-axis refers to the percentage of gene pair overlap.

#### Comparison between gene pair overlap and gene overlap algorithm

**Figure 3:**
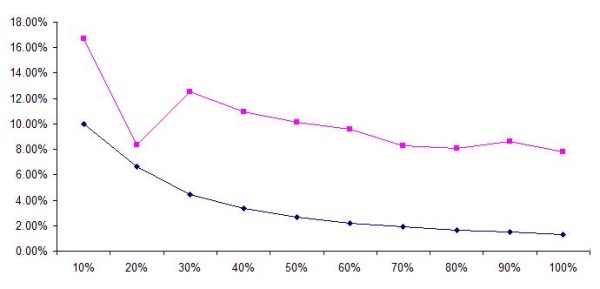
**Comparison between gene/gene pair overlap algorithm with the LCS algorithm**. Image comparing the overlap between the gene overlap algorithm (blue) and gene pair overlap (purple) with the LCS algorithm. The image shows that the gene pair overlap algorithm is generally better than that of the gene overlap algorithm.

Our final analysis involves taking the LCS algorithm as the reference algorithm and comparing the results of the gene pair overlap and gene overlap approaches with it. The results are reproduced in Figure 3. The purple graph refers to the graph for gene pair overlap while the blue graph refers to the graph for gene overlap. The x-axis refers to the top (10%, 20%, etc) matching pairs (ranked based on the size of gene overlap or gene-pair overlap respectively from the two algorithms) that are obtained. The y-axis refers to the percentage of overlap of the pathway pairs between LCS and the gene pair overlap or gene overlap algorithm. (For example, for the first 10% of pathway pairs for the gene pair overlap algorithm, there is a 17% overlap with that of the LCS.)

The graph shows the gradual decreasing of percentage overlap, with the gene pair overlap graph always staying above the gene overlap graph. This shows that the gene pair overlap algorithm generally has more accurate results than the gene overlap algorithm (which fits into our earlier postulation).

#### Conclusion

Therefore, our LCS technique achieves superior performance (accuracy and completeness) compared to the method of matching pathways according to their gene overlap. We can safely conclude that the LCS algorithm is most suitable for our purpose.

### Database Comprehensiveness

This section conducts an independent audit on the comprehensiveness of individual pathway databases. We use two metrics to determine the comprehensiveness of individual pathway databases. The first metric known as the "Pathway Comprehensive Score" determines the comprehensiveness of pathways within databases. The second metric is known as the "Gene Pair Coverage Score" and it determines how comprehensively each database covers the number of unique gene pairs within the databases. This is accomplished by dividing the number of gene pairs within each database by the total number of gene pairs within our database. We will elaborate further within this section.

The "Pathway Comprehensive Score" metric first counts the total number of unique pathways present within the three databases (Ingenuity, KEGG and Wikipathways). A score for each database is next calculated by dividing the number of pathways a database hosts by the total number of unique pathways. A score of 0 indicates that the database hosts nil pathways while a score of 1 indicates it hosts all the pathways. Thus databases having a small number of very large pathways will score low on this metric because it is missing out many pathways completely.

**Figure 4:**
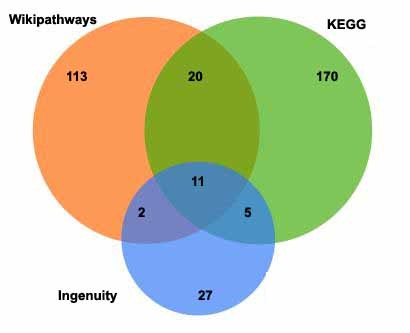
**Comparison of Database Comprehensiveness**. Comparison of Database Comprehensiveness.

KEGG achieves the highest score of 0.59. This is followed by Wikipathways (0.42) and Ingenuity (0.13). This short study indicates that KEGG Pathways remains the most comprehensive of all databases in terms of number of pathways contained. This is illustrated by a Venn diagram in Figure 4.

The second metric known as the "Gene Pair Coverage Score" calculates the percentage of gene pairs each individual database has. It first counts the total number of unique gene pairs present within the three databases (Ingenuity, KEGG and Wikipathways). A score for each database is next calculated by dividing the number of gene pairs a database hosts by the total number of unique gene pairs. Here, KEGG achieves the highest score of 0.65. This is followed by Wikipathways (0.27) and Ingenuity (0.16). In interpreting these numbers, it is important to bear in mind that KEGG contains both regulatory pathways and metabolic pathways. The latter pathways are not the focus of Wikipathways and Ingenuity. As a result, KEGG contains many gene pairs which catalyze adjacent steps in metabolic pathways that are not found in Wikipathways and Ingenuity.

### Database Compatibility

The two preceding sections demonstrate the inadequacy of using only one selected database for data analysis. Cross-database queries are the intuitive solution to harness the required information across these databases. However incompatibilities between different databases makes cross-database accesses extremely challenging to execute. We investigate these incompatibilities across different databases and present them here.

#### Incompatible methods of data access

Different databases use different methods of data access. Some databases only allow data to be downloaded via web access. Others provide flexible access to their databases through their API.

A lot of human intervention is required to download the required information for databases with no public API. This creates tedious challenges for software to obtain information from such databases.

For databases whose API is public, there is no guarantee that all such API would use the same programming languages. This causes developers to incorporate clumsy wrappers within their applications to adhere to the API of the databases.

#### Incompatible data formats

All databases release their pathway information via some non-standard graphical format. Such a graphical representation is useful for visual manual analysis. However, it is inconvenient for large-scale computational analysis.

Some repositories do release their data in formats such as their proprietary markup languages or API data structures. These are more convenient for large-scale analysis. Indeed there are some efforts to make data exchange formats compatible with one another. An example of such an effort is BioPax [16]. We have carried out a survey of the data formats used by popular biological databases and found many who have not adopted such standardized formats and have continued using their own proprietary data formats. These findings are summarized in the Table 8.

**Table 8:** Table comparing different data formats supported by different databases.

Comparison of Data Formats					
**Database**	**Pictorial**	**Proprietary**	**API/Data Dump**	**BioPax**	**Biopax Format**

KEGG	Yes	Yes	Yes	No	NA

Ingenuity	Yes	No	No	No	NA

Wikipathways	Yes	Yes	No	No	NA

Reactome	Yes	Yes	Yes	Yes	Lvl 3

HumanCyc	Yes	Yes	No	Yes	Lvl 2

BioCyc	Yes	No	Yes	Yes	Lvl 3

Pathway Commons	Yes	Yes	Yes	Yes	Lvl 3

We point out that Biopax has since updated its data format three times, known as Level 1, Level 2 and Level 3.

It has been demonstrated that JSON (as compared to XML) uses significantly less server computational resources and is capable of delivering content within a much shorter time in [17]. E.g., one of the experiments in [17] showed that the total time to access 100,000 objects took 78.26 seconds using JSON while taking 75.77 minutes with XML. The same experiment shows that the average server utilization when using JSON was 13% compared with 45% for XML. Other references [18] have also estimated that JSON parses data up to 100 times faster then XML in modern browsers.

We would like to highlight the fact that although compression techniques are available to reduce the amount of data to be transferred to 2-23% of their original size [19], these techniques will inadvertently complicate the architecture and increase client side processing [19]. The same review paper also surveyed and compared XML compression techniques and concluded that there is still a lack of state-of-the-art XML compression techniques that are stable, efficient and stable [19].

Hence for the above reasons, the flat xml file formats used by BioPax might not be the best choice for large-scale computational manipulation especially when dealing with large-scale data of tens of thousands of genes over hundreds of pathways. We have therefore opted for using JSON as the data exchange format. This lack of a consistent data format means that different databases use different formats to represent their data. Hence dedicated codes have to be written to parse, understand and integrate data from each individual database. Furthermore, note that the KEGG no longer supports the BioPax format. From the WWW, even the KEGG data in BioPax Level 1 (from January 2006) are no longer available. The current method of doing so is to convert the KEGG KGML data format first into PID (Pathway Interaction Database) format and then converting it to BioPax Level 2.

#### Incompatible molecular representations

Different repositories assign different naming conventions to their pathway nodes. These nodes can be described as proteins, genes or symbols depicting protein families. For example, KEGG describes most of their elements as genes, Ingenuity describes them as proteins, while wikipathways uses a combination of both.

Hence it is possible to miss crucial genetic relationships because of such inconsistent representation. To obtain all relationships represented within pathways, algorithms are required to convert all nodes to a common representation.

#### Incompatible pathway names

Common biological pathways in different databases are often given names with limited indication of how pathways are related to one another. For instance, KEGG may refer to a pathway as "Wnt signaling and pluripotency" and the Wikipathways might refer to it simply as "Wnt signaling". Other than the fact that both pathways have the common terms "Wnt signaling", there is no way of knowing that the "Wnt signaling" pathway is a subset of the "Wnt signaling and pluripotency" pathway other than through human intervention.

This makes it difficult to determine pathways that refer to similar biological processes (albeit sporting different pathway names). It is difficult to match and compare similar pathways across different repositories.

#### Inconsistent data

Sometimes there is contradictory information across different biological sources. When such a scenario occurs, the algorithm has to decide which contradictory information to discard and which to keep. For instance within the KEGG's Cell Cycle Pathway, gene RB1 activates gene TFDP1. Ingenuity's Cell Cycle Pathway however states that gene RB1 inhibits gene TFDP1.

### Construction and content

In view of these issues mentioned above, we provide a single common API to access the different databases. This common API works in this manner: A local database serves as a cache, storing data from the other repositories. Requests for information from the different repositories are directed to this cache to obtain the required information. To ensure that our interface is always kept up to date, automatic incremental updates are run periodically to extract the latest information from the different repositories. This process creates a unified interface for the different databases, as well as a unified database where graphs of the same pathway are merged.

The database cache currently stores a total of 397 gene pathways, 21,314 genes and 60,900 gene pairs. From this API, access to pathways from both the integrated and the individual sources are provided. Further details of this interface can be found at [20]. The API consists of the following modules:

+ Pathway Formalization: Key features within pathways

+ Database Cache: How we store, extract and update the data

+ API Implementation: Short specification of the API

### Pathway Formalization: Key features within pathways

Pathway databases supply many informative features that are useful for the purposes that these databases were originally intended for. However, for use in gene expression analysis algorithms such as ORA, FCS and GSEA it is sufficient to capture only two key features in these pathway databases.

One feature defines all the genes within the pathway while the other defines gene-gene relationship within the pathway. Here we only consider two relationships between genes: activation and inhibition. (Gene relationships in metabolic pathways are formalized in the same manner based on how they catalyze adjacent steps within the pathway. For metabolic pathways, relationships between adjacent proteins are indicated as neutral, meaning neither activating or inhibiting.) This formalization helps organize and streamline information within pathways.

**Figure 5:**
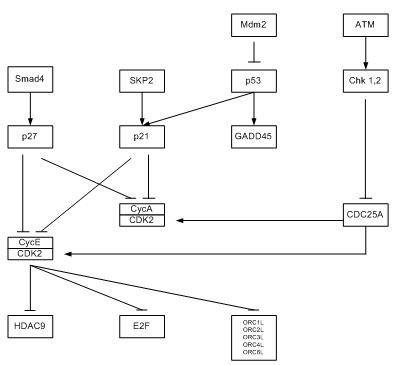
**Sample KEGG Pathway**. A short and sample of the KEGG Pathway simplified from an original KEGG pathway.

**Figure 6:**
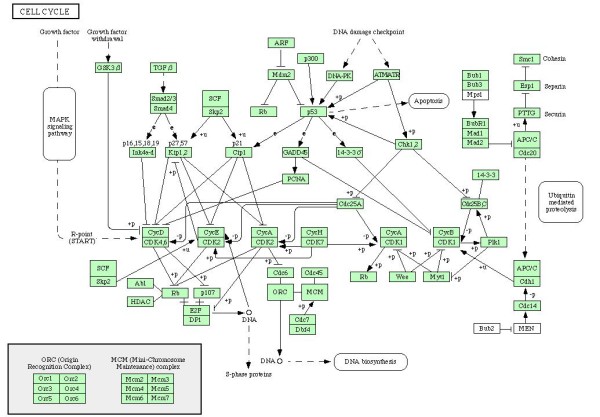
**The original KEGG Pathway**. The original KEGG pathway.

For illustration, we redraw a KEGG pathway in Figure 5. The original pathway is in Figure 6. The component depicting genes within a pathway refers to the individual genes MDM2, TP53, etc. The other component depiciting gene-gene relationships refer to relationships (eg MDM2 inhibits P53, ATM activates CHK1) in the pathway diagram.

#### Representing Genes within a pathway

As mentioned earlier, one of the inconsistencies across different databases is the inconsistent usage of proteins, genes or protein lists within pathway data. To address this issue, all gene or protein representations are converted to their corresponding NCBI Gene ID. The NCBI Gene ID is obtained by issueing and parsing the results of the query:

Webquery 1

http://www.ncbi.nlm.nih.gov/entrez/query.fcgi?db=gene&cmd=search&term=Y+homo+sapiens

The symbol *Y *refers to the gene name. Executing this query iteratively across all the genes/proteins within the pathway provides us with the Gene IDs within the pathway. This common terminology reconciles gene naming inconsistencies across the different repositories.

#### Representing Genes-Gene Relationships within a pathway

**Figure 7:**
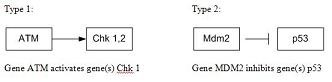
**Shows two sample relationships**. The inhibiting and activating gene-gene relationship. The left relationship shows an activating relationship between ATM and Chk 1,2 while the right relationship shows an inhibiting relationship between MDM2 and P53.

There are only two types of relationships present between genes: inhibition and activation. These two relationships are illustrated in Figure 7 where we see ATM activating CHK1, CHK2 and MDM2 inhibiting p53.

By constructing such inhibitor-inhibitee/activator-activatee relationships, investigators explicitly know the exact relationship of genes within pathways. This allows them to analyze the adherence of these relationships in their experimental data.

### Database Cache: How we store, extract and update the data

#### Data Storage

We maintain a database cache to store information from the other pathway repositories. To ensure fast response to users, all queries submitted are directed to this database cache. Our database cache is kept up to date with a set of automated scripts written to do periodic incremental updates from the other databases.

#### Data Extraction: Wikipathways

Data from wikipathways are publicly available via their proprietary file format known as the GPML format [21]. Hence we first obtain the pathway IDs of all the pathways present within the wikipathways database. The next step involves iterating through these ids to obtain the GPML file associated to each pathway ID. The final step parses the GPML format to obtain the pathway genes and associations. All pathways within wikipathways are obtained by issuing and parsing the query:

**Webquery 2 **http://www.wikipathways.org/index.php/Special:BrowsePathwaysPage

The corresponding GPML file for each pathway is obtained with this query:

**Webquery 3 **http://www.wikipathways.org//wpi/batchDownload.php?species=Homo%20sapiens&fileType=gpml&tag_excl=Curation:Tutorial

Here *X *refers to the name of the pathway.

The GPML format is designed towards the visual display of pathway information. Hence it contains detailed coordinate information about the spatial location of genes and arrows/t-bars (which depict activating/inhibiting relationships). Yet how these genes are related is not described in the GPML specifications. A parser is therefore needed to understand these spatial descriptions and extract the relevant genes and associations. The different components of the parser are:

+ **Gene Extraction**: Extraction of genes from the GPML file requires the identification all occurrences of the GPML DOM attribute name: "DataNode". This enables the parser to obtain the Gene Name, Gene NCBI ID and the spatial coordinate locations associated to this datanode.

+ **Spatial Clustering**: Activating/inhibiting relationships are described across gene clusters spatially. Therefore the genes have to be spatially clustered to determine spatially the activator/activatee or inhibitor/inhibitee relationships.

**Figure 8:**
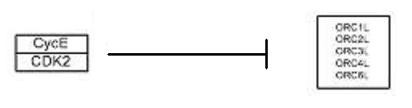
**Spatial relationships between genes**. The appearance of spatial relationships. Here the genes CycE and CDK2 are seen inhibiting the family of ORC genes.

An example is reproduced in Figure 8. Here the genes CDK2, CYCE inhibits the entire cluster of ORC genes. Hence we need to group the genes CDK2, CYCE together as one cluster, and the ORC genes as another.

Using the coordinates from the genes obtained above, a nearest neighbour technique is employed to organize the genes into their respective clusters. Basically this nearest neighbour algorithm groups genes together if their distance apart is below a threshold (empirically determined as 100 pixels).

+ **Relationship Extraction**: Relationships within the GPML files are represented by the attribute keywords: "Arrow" for activating and "T-Bar" for inhibiting. These attributes provide their spatial coordinate information of activating and inhibiting relatioships. The challenge here is associating the correct gene clusters to each relationship.

By representing relationships as a straight line, this relationship line in the spatial space is extended until it intersects with the nearest gene clusters on both sides of it. This technique assigns the activator/activatee or the inhibitor/inhibitee gene clusters to both sides of the relationship.

For metabolic pathways (because the gene relationship is neither activating or inhibiting), the GPML attribute keyword is simply a "Solid" line attribute. In such instances, the relationship type attribute to the gene pair would be "neutral".

#### Data Extraction: KEGG

Data is obtained from KEGG via a series of API calls and processing the data (SOAP format) returned. An API call is issued to obtain all the pathways first. This returns all the relevant pathway IDs stored within KEGG. Separate API calls are made for each pathway ID to obtain the genes and gene pairs present for each specific pathway.

The API call to obtain all the pathways for homo sapiens is:

**API 1 ***serv.list_-_pathways("hsa")*

where "serv" refers to the created wdsl object to communicate with KEGG and hsa refers to the "homo sapiens species".

The API call for gene and gene pair extraction from a KEGG pathway is:

**API 2 ***serv.get_-_genes_-_by_-_pathway(X)*

**API 3 ***serv.get_-_element_-_relations_-_by_-_pathway(X)*

where × refers to the pathway ID within KEGG.

#### Data Extraction: Ingenuity

Most pathway information available from Ingenuity is in a pictorial format. This forces pathway data extraction to be done manually, an extremely painful and time consuming process.

### Data Updates

An expiry date is assigned to all information stored within our database cache. Upon reaching the expiry date, scripts are triggered to run, automatically extracting information from the reference databases (KEGG and Wikipathways) and populating it into our database cache. (Note that in the event where new pathways are extracted, they are first matched by the LCS algorithm and stored on a temporary database before the manual process of scanning through the pathway pairs).

### API Implementation: Short specification of the API

The API was written in PHP, and data transfer in JSON format. We have chosen JSON over SOAP or XML because:

+ JSON is lighter in weight, transmitting less information over the internet. Client applications therefore executes faster.

+ JSON has the ability to easily represent most general data structures such as records, lists and trees.

+ With SOAP or XML, dedicated parsers are always required on the client. JSON is innately supported by most programming languages, eliminating the need for client parsers.

### Utility

The implemented functions of the API include:

+ GetDatabase: Returns all repositories supported by our API. No parameters are required for this function. The usage example is: http://www.pathwayapi.com/api/API_GetDatabase.php and the sample results returned is: ["KEGG","Ingenuity","Wiki"].

+ GetGene: Returns the NCBI GeneID of the gene. This function takes the name of the gene as the parameter. An usage example is:

http://www.pathwayapi.com/api/API_GetGeneID.php?SearchGene=MDM1. The format returned is: [["MDM1","252867"],["MDM1","56890"]]. In this case, there are two separate gene ids that are returned.

+ GetDBPathways: Returns the all pathway names and IDs of a specific repository. Only the database name needs to be submitted to the function. For instance,

http://www.pathwayapi.com/api/API_GetDBPathways.php?DatabaseName=KEGG. Here, the following will be returned: [kegg{"1":{"DatabaseName":"KEGG","PathwayName":"Glycolysis Gluconeogenesis - Homo sapiens (human)"}, "2":{"DatabaseName":"KEGG", "PathwayName":"Citrate cycle (TCA cycle) - Homo sapiens (human)"}, etc...] where "1" refers to the Pathway ID, "KEGG" refers to the name of the database and "Glycolysis Gluconeogenesis..." refers to the name of the pathway.

+ GetPathway: Returns the pathway ID of a specific pathway of a repository. Posting the name of the pathway in this manner: http://www.pathwayapi.com/api/API_GetPathway.php?Pathway=Apoptosis will return the jason format like: [["Apoptosis - Homo sapiens (human)","KEGG","140"], ["Apoptosis Signaling","Ingenuity","210"]].

In this instance, this implies that there are at least two pathways with the "Apoptosis" keyword witin their pathway names. The two pathways occurs in the KEGG databases and in the Ingenuity databases. The pathway id associated to each is 140 and 210 respectively.

+ GetPathwayGenes: Returns all the GeneID of a specific pathway of a repository. Providing the pathway ID to this function will return you the genes within this pathway in this manner: http://www.pathwayapi.com/api/API_GetPathwayGenes.php?Pathway = 7 Resulting in: ["231":"AKR1B1","2538":"G6PC","2548":"GAA","2582":"GALE"] where "231" refers to the gene ID and "AKR1B1" refers to the name of the gene.

+ GetGenePathways: Returns all the pathways which a gene occurs. In the opposite note, this function will return all the pathways which a supplied gene occurs in. http://www.pathwayapi.com/api/API_GetGenePathways.php?SearchGene = 7157 We obtain the following database pathway pairs: ["128":"MAPK signaling pathway - Homo sapiens (human)","134":"Cell cycle - Homo sapiens (human)","135":"p53 signaling pathway - Homo sapiens (human)"] In this example, "128" refers to the Pathway ID and "MAPK signaling pathway - Homo sapiens (human)" refers to the name of the pathway.

+ GetPathwayInteractions: Returns all interactions within a pathway of a database. Passing in the ID of the pathway, the API will return all the interactions within the pathway. http://www.pathwayapi.com/api/API_GetPathwayInteractions.php?Pathway = 7 will result in [["231","AKR1B1","2584","GALK1","Activate"],["231","AKR1B1","2585","GALK2","Activate"]] In the example above: "231" and "2584" refers to the IDs of the gene pair "AKR1B1" and "GALK1" refers to the corresponding genes of the ID.

+ GetPathwayDiff: Get the differences in genes and gene interactions across pathways. This function requires you to supply the IDs of the two pathways you wish to check on the difference in. The call below shows the difference in genes and gene interactions between pathway 7 and pathway 8. http://www.pathwayapi.com/api/API_GetPathwayDiff.php?Pathway1 = 7&Pathway2 = 8

This gives the following results where: [["AKR1B1","G6PC","GAA","GALE","GALK1"], ["ALDH2","ALDH3A1"], ["AKR1B1_GALK1","AKR1B1_GALK2","AKR1B1_GLA"],[]] Where ["AKR1B1","G6PC","GAA","GALE","GALK1"] refers to the genes within pathway 7 not in pathway 8.

["ALDH2","ALDH3A1"] refers to the genes within pathway 8 not in pathway 7.

["AKR1B1_GALK1","AKR1B1_GALK2","AKR1B1_GLA"] refers to the gene interactions within pathway 7 not in pathway 8.

[] refers to the gene interactions within pathway 8 not in pathway 7. This set is empty because all interactions in pathway 8 are in pathway 7.

## Conclusions

It is widely accepted that analyzing microarray experiments with biological information provides biological inferences of a greater detail. Examples of such analysis are [22-24].

However, such techniques run into issues if the data source used is not consistent or comprehensive. For example, using the same technique on a different database yields a differing analysis result.

Faced with such an issue, the solution is to integrate biological information across different data sources to obtain a more wholesome analysis. Yet the incompatibility of the different data sources renders this option extremely challenging.

Furthermore, we investigated and discovered low levels of consistency, comprehensiveness and compatibility among three popular pathway databases (KEGG, Ingenuity and Wikipathways).

Our strategy of addressing this issue was to create an API (freely available) which gives researchers access to various pathway databases of their choice as well as to an integrated database. This integrated database resolves various incompatibility issues between databases such as:

1. Incompatible methods of data access

2. Incompatible data formats

3. Incompatible molecular representations

4. Incompatible pathway names

However we understand the limitations faced by such systems. For instance, in the event that two databases provide conflicting definitions of the same pathway, both pieces of conflicting definitions will be included within the pathway information itself. The integrated database is also more comprehensive because it is the union of the data sources. Every gene/edge in any of the three data sources is also in the integrated database. Furthermore, the integrated database is equipped with a API to allow the user to conveniently identify inconsistencies and to resolve them in accordance to his specific application needs. To ensure fast responsiveness, API connections are made towards a central unified database which keeps a cached copy of the records of the other databases. To make certain that the cached entries are always kept up to date, entries from the cache are flushed periodically and automatically updated again for the reference databases wikipathways and KEGG. As data from Ingenuity is obtained in a manual fashion, updates from Ingenuity can only be achieved laboriously by curators importing new data physically from Ingenuity. Note that data from Ingenuity will not be released.

There are many efforts on the aggregation of pathways data (like Reactome [25], PathCase [26,27] and MappFinder [28]). There are also many tools to explore, edit and export biological pathways (such as GenMapp [29], BioCyc [30], PathVisio [31], Cytoscape [32]).

However manipulation of pathways in these earlier works still relies heavily on human intervention with little provision for programming interfaces. Indeed projects like Cytoscape and Pathcase have very sophisticated GUI visualization tools to help researchers manipulate pathways. Such visualization tools are impractical when you are required to analyze thousands of genes across hundreds of pathways for each microarray experiment. The nearest to a programming interface was the provision of a AQI (Application Query Interface) [26] where users can recall predefined queries using a web interface. Yet the scope of such queries remains limited and insufficient.

Two of the projects that are similar to this work are Pathway Commons [33] and BioWarehouse [34]. BioWarehouse is an open-source software environment and is often used for integrating a set of biological databases into a single physical database management system for data management, mining, and exploration. They are able to do so by first deciding on a standard database schema and having database loaders to import certain pathway database formats (eg BioPax) into their environment. Being more of a software environment, they act more as a tool to give researchers a easy way to merge data whereas we focus on allowing researchers easy access to the data and on analysing the completeness and consistency of the data. PathwayCommons is an aggregated pathway database as well. Most of the databases they host support the BioPax data format. However they do not host any of the three databases that we aggregate. Thus our unified database and API are very much complementary to the contributions Pathway Commons has done.

One issue we have with most data aggregators is their lack of explanation on how their data is kept updated. For instance, little mention is made on how the aggregated data is updated from the various repositories. In fact this issue is acknowledged in [26]. Here we set an expiry date for every data entry and once it expires, automated scripts are fired off to extract data from the data sources and populate them within our database cache.

Our final point deals with the aggregator's inaction to develop integrated pathway data from their diverse data sources. By standardizing gene references and key features within pathways, we have the ability to integrate similar pathways together. As a result our integrated pathways are more comprehensive. Contrasting to prior available methods, researchers can easily use our API to obtain data for each pathway either from the integrated database or from a specific database of their choice. This gives researchers a straightforward mechanism for incorporating pathway information into their microarray analysis.

### Availability and requirements

**Figure 9:**
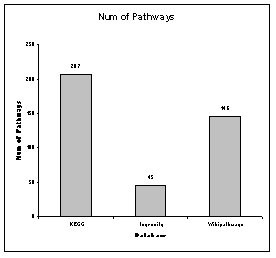
**Number of pathways in each database**. The number of pathways in each database.

**Figure 10:**
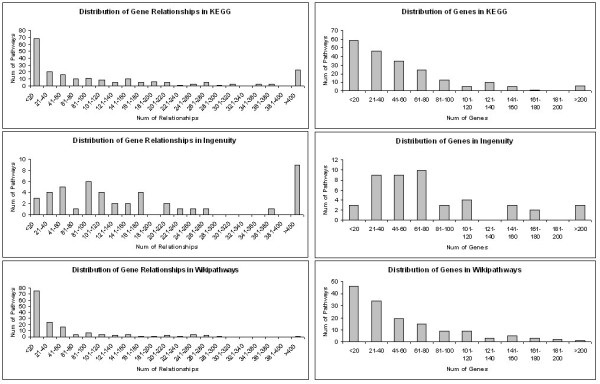
**Distribution of pathway sizes in terms of genes and gene pairs**. The left column shows the distribution of gene pairs within the three databases while the right column shows the distribution of genes.

The database is available at http://www.pathwayapi.com. Data from the sources can be accessed via the API as described. In addition, the data will be available for download as a CSV or SQL file. The database schema and instructions on their usage are stated in the downloaded file. All information will be provided free of charge. Information from Ingenuity will however not be released as it is proprietary (unless the requester has a valid subscription to Ingenuity). The latest number of pathways in each database are shown in Figure 9 while Figure 10 shows the distribution of pathway sizes in terms of genes and gene pairs. The recommended requirements are: 1 Mbps internet connection, 1GHz Processor, 512MB Memory.

## Authors' contributions

DS developed the software and database. DD provided the data from Ingenuity and pathway analysis. DS and LW wrote the manuscript. All authors contributed to design of analytical algorithms. All authors read and approved the final manuscript. This research is supported in part by an A*STAR AIP scholarship (DS), a A*STAR grant SERC 072 101 0016 (LW, DD).
